# Diagnostics of Concrete and Steel in Elements of an Historic Reinforced Concrete Structure

**DOI:** 10.3390/ma14020306

**Published:** 2021-01-08

**Authors:** Paweł Tworzewski, Wioletta Raczkiewicz, Przemysław Czapik, Justyna Tworzewska

**Affiliations:** Faculty of Civil Engineering and Architecture, Kielce University of Technology, Al. Tysiąclecia Państwa Polskiego 7, 25-314 Kielce, Poland; ptworzewski@tu.kielce.pl (P.T.); p.czapik@tu.kielce.pl (P.C.); j.tworzewska@tu.kielce.pl (J.T.)

**Keywords:** reinforced concrete, diagnostic testing, corrosion, carbonation, galvanostatic pulse method, phase composition analysis, X-ray analysis, thermal analysis

## Abstract

Existing buildings, especially historical buildings, require periodic or situational diagnostic tests. If a building is in use, advanced non-destructive or semi-destructive methods should be used. In the diagnosis of reinforced concrete structures, tests allowing to assess the condition of the reinforcement and concrete cover are particularly important. The article presents non-destructive and semi-destructive research methods that are used for such tests, as well as the results of tests performed for selected elements of a historic water tower structure. The assessment of the corrosion risk of the reinforcement was carried out with the use of a semi-destructive galvanostatic pulse method. The protective properties of the concrete cover were checked by the carbonation test and the phase analysis of the concrete. X-ray diffractometry and thermal analysis methods were used for this. In order to determine the position of the reinforcement and to estimate the concrete cover thickness distribution, a ferromagnetic detection system was used. The comprehensive application of several test methods allowed mutual verification of the results and the drawing of reliable conclusions. The results indicated a very poor state of the reinforcement, loss in the depth of cover and sulphate corrosion.

## 1. Introduction

Periodic inspections of existing buildings, as well as renovation or modernisation works and even planned demolition works, require appropriate diagnostic tests [1,2,3]. In standard periodic inspections aimed at assessing the technical condition of the facility, the procedures for carrying out such tests are governed by relevant regulations, e.g., construction law in Poland [4]. However, in other cases, especially when it comes to examinations after a construction failure or due to the desire to change the existing utility function of the facility (despite the existing general recommendations), it is necessary to approach a given case individually and prepare an original program of diagnostic tests. Particular attention should be paid to the research of historic buildings, in which any invasive work interfering with the structure of the elements is often prohibited [2,3,4,5,6,7]. In such situations, it is usually necessary to use non-destructive or semi-destructive methods [1,3,8].

The significant and rapid development of technology in the field of measurement methods has recently contributed to the use of tools in construction diagnostics that allow destructive tests of elements and specimens as well as measurements with methods that are non-destructive or semi-destructive in their effects on building structures [1,8,9,10,11]. Additionally, measurements can be made with ever-greater precision. Due to the multitude of test methods that can be used, it is important to choose the right measurement method appropriate to the situation. If possible, it is worth verifying the obtained results with several methods. This is very important in obtaining reliable results, minimising measurement errors and reaching accurate conclusions.

Carefully considered diagnostic tests allow verification of the data contained in historic project documentation or obtained on the basis of an analytical model [2,3,7,12]. Based on appropriately selected and performed diagnostic tests, it is possible to develop reliable expertise indicating the causes of possible damage. Recommendations regarding the scope, order and manner of repair and upgrading work can be made.

However, not only should a well-prepared program of diagnostic tests account for an assessment of the existing condition, it should also provide a forecast for the durability of the structure in the future. This applies in particular to load-bearing structures operated in unfavourable environmental conditions, in which the design assumptions for ensuring adequate durability were incorrect [1,7,8,9,10,11,12,13,14,15,16]. In the article [16], the authors pay special attention to the chemical tests of the material taken from the structure (usually small samples of the material obtained while performing semi-destructive tests), which allows assessment of the chemical condition of the elements in the context of protection against corrosion during its future operation. The authors believe that the decisive factor in the possibility of effective and durable repair of a corroded reinforced concrete structure is its chemical condition.

The comprehensive and systematised classification of test methods for the diagnosis of concrete structures can be found in [17].

In the article, the authors also provided information on external and internal factors influencing the degradation of the structure, the degree of their impact and the optimal selection of research methods (destructive, non-destructive and semi-destructive). Diagnostic tests can be divided into tests aimed at estimating the load-bearing capacity of structure elements and strength parameters of materials (very often destructive or semi-destructive tests) and tests aimed at assessing the durability of elements and the entire structure. The authors took into account the usefulness of using various methods of assessing the durability of structures depending on the degradation mechanisms and their impact on durability. Non-destructive methods (NDT) were analysed, i.e., those that do not violate the integrity of the structure in any way, and semi-destructive methods (SDT), i.e., those that require material sampling or that slightly disturb the integrity of the structure. The authors proposed the classification of physicochemical and biological diagnostic methods to assess the durability of RC structures and presented the research equipment [17].

In the case of reinforced concrete structures, diagnostic tests primarily concern the assessment of the state of reinforcement and the concrete cover, which directly determines the durability of the structure [1,10,13,14,15,18,19,20].

In reinforced concrete structures, concrete (if it is tight enough, was made according to the appropriate recipe and has the right thickness) is the basic and best protection for the reinforcement [10,11,13,21,22]. The protective role of concrete is related to its alkaline reaction (pH ≈ 12.5–13.5), giving the effect of passivation at the interface between concrete and steel (formation of a micro-layer with very low ionic conductivity), which for all practical purposes prevents corrosion of the bars. Unfortunately, because of the negative long-term effects of physical and chemical factors of the external environment as well as to possible mechanical damage, the protective qualities of the concrete cover deteriorate.

Most often the deterioration is the effect of carbon dioxide in the air and changes in temperature and humidity of the environment, which leads to gradual carbonation of concrete and directly affects the loss of the protective properties of the cover in relation to the reinforcement [10,11,21,22,23,24].

As a result of carbonation, the pH of the concrete gradually decreases, and the areas of neutralised concrete extend further and further into the cover, eventually reaching the passive layer. In areas where the pH of the concrete drops below ~11.8, the surface of the reinforcing steel is depassivated, which directly leads to the initiation of electrochemical corrosion of the reinforcement.

On the other hand, in the case of seaside facilities located in the coastal zone (port and tourist buildings), exposed either to the direct influence of sea salt or to the so-called salt mist, as well as road structures (bridges, viaducts, flyovers), concrete surfaces, parking lots and open garages, due to the use of de-icing agents containing NaCl, the cause of degradation is the action of chlorides [10,21,23,24,25,26,27]. Sulphates that degrade concrete are also dangerous [28,29].

On the other hand, mechanical damage to the concrete cover (caused by accidents or minor damage accompanying the use of the facility), although visible to the naked eye in the form of cracks and defects, paradoxically often does not indicate an advanced process of structural degradation [30].

Nevertheless, the assessment of the load-bearing capacity of structural elements is a very important factor in the diagnostics of buildings. For this purpose, computational and experimental models are often used [31,32,33]. It is especially important in the case of unusual load, such as high temperature caused by fire [34]. In recent years, neural networks have been used for diagnostics of structural elements of buildings [35,36].

Usually, diagnostics of reinforced concrete structure elements is carried out in several ways. One of them concerns the measurements of the thickness and continuity of the concrete cover and the location of the reinforcement. The second are electrochemical, non-destructive or semi-destructive tests, which indirectly allow one to determine and forecast the degree of concrete and steel corrosion advancement. Third is the testing of the material, i.e., fragments of concrete cover taken from the structure, on the basis of which the chemical composition of concrete and its microstructure are determined. Computational and experimental models are also used to assess the load-bearing capacity of structural elements.

The applied research methods are quite known in the diagnostics of reinforced concrete structures. However, often the tests are performed selectively—they are limited to one type of method, which provides limited information. The authors would like to point out that only the knowledge and combination of several methods allows for the preparation of an appropriate research plan and a full assessment of the condition of the structure, especially if it concerns historic objects, in the diagnostics of which it is only possible to use non-destructive or semi-destructive methods, as in the described example. Additionally, the authors would like to draw attention to the measurement techniques used. While the ferromagnetic method used in the research can be successfully replaced by other methods (which are described in more detail in this chapter), the galvanostatic pulse method seems to be the most precise and easy to use compared to other commonly used electrochemical methods. X-ray analysis and phase composition analysis are also noteworthy as techniques allowing for very precise recognition of the effects of carbonation.

## 2. Materials and Methods

The thickness of the concrete cover is one of the key factors determining the durability of reinforced concrete structures susceptible to aggressive external environment, because it determines the speed of penetration of aggressive substances that can damage the passive layer and initiate corrosion of the reinforcement [10,11,13,15,29].

There are many test methods that allow determining the thickness of the cover and locating the reinforcement bars, their diameter and mutual position. Currently, a number of the methods in use are based on changes in electromagnetic waves penetrating through the tested reinforced concrete element medium. The tests can be performed using electromagnetic, radiological, ultrasonic, or radar methods. The precision of the obtained results as well as the effectiveness and efficiency of the research as a function of time depend on the research method used.

One of the most accurate among those mentioned above is the radiological (radiographic) method, which uses ionising radiation (X-rays or gamma rays) [37,38,39,40]. Because of this, it is possible to determine the geometrical parameters of the reinforcement with an accuracy of 5%. It is the best method in terms of accuracy that allows locating the reinforcement and its parameters in complex reinforced concrete elements. However, its application requires the use of complex equipment and protection against harmful radiation. The long duration of the research also contributes to the low popularity of this type of research.

A more widely used alternative to radiographic methods are electromagnetic methods [3,37], which use the phenomenon of electromagnetic field changes according to the ferromagnetics (steel) system in a reinforced concrete element [1,41,42,43]. Modern devices used to perform tests with this method are easy to use (intuitive). The analysis of the scanned reinforcement image is also not complicated [44,45]. Unfortunately, this type of research is not the most accurate and is susceptible to the influence of many factors.

The radar method can also be used to locate reinforcement in concrete. It uses the phenomenon of the penetration and reflection of an electromagnetic wave (in the range of 100 MHz–2 GHz) through a medium (reinforced concrete element) which, depending on the medium material type (concrete or steel), the wave penetrates into depth or is scattered or reflected [46,47,48,49,50]. In the case of the currently used equipment, the method of conducting the research itself is similar to the electromagnetic method, but the interpretation of the results requires more knowledge. Fortunately, the development of computer software greatly facilitates the operation and analysis of the obtained phalograms. As in the case of the previous method, this method is also susceptible to the influence of many factors that may disturb or prevent the analysis of the obtained results.

Impact-echo [51] and impulse response [52] methods, which rely on the analysis of the flow time of a mechanically excited wave in the tested medium (concrete), can also be used to assess the continuity of the reinforcement. The acoustic emission (AE) method is also more and more widely used [53,54,55]. It consists of the analysis of differences in the acoustic wave propagation speed in reinforced concrete. However, these methods do not allow for measuring the diameter of the bars or for the precise measurement of the reinforcement cover thickness.

In most cases, electromagnetic methods are effective and sufficient. In the tests described in this article, the PS 200 Hilti (Schaan, Liechtenstein) scanner was used to locate the reinforcement and determine the cover parameters. This device has been on the market for a long time. It enables easy and quick measurement and subsequent analysis of the obtained results. The performed measurements allow one to estimate the cover thickness and the diameter of the reinforcement. The ability to perform surface scans and combine them is particularly useful. As mentioned earlier, this is not an accurate method, and any magnetic materials in the concrete, such as tie wire or other ferrous particles, as well as reinforcement corrosion products, will distort the results. However, it is still the fastest method of reinforcement detection and in most cases it is sufficient in the diagnosis of reinforced concrete elements [15].

Some of the mentioned methods allow the detection of defects and discontinuities of the material that are not visible on the surface. The method of acoustic emission allows, among others, the recording of acoustic signals caused by defects in the microstructure of the material during the use of the structure [55]. The advantage of this method is its sensitivity and, as a result, its ability to determine various destructive processes that took place in the tested element at a given time.

On the other hand, defects in the form of scratches and cracks visible on the concrete surface can be diagnosed quite simply with the use of practical handy tools [1]. To measure the width, depth and activity of cracks, one can use feeler gauges or templates (paper or plastic), as well as a Brinell magnifier with a scale (Dioptra Turnov, Czech Republic) (optical instrument with a magnification of 8 to 40 times, with a scale of 0.05 mm or 0.1 mm). Mechanical feeler gauges (drawing gauges, gauges or crack gauges) allow one to measure the width of the crack as well as monitor displacements in one, two, or even three directions. In complicated cases where access to the crack is difficult, the problem can be overcome by the use of microcameras, which are an electronic version of a magnifier with a scale. Endoscopes (rigid—boroscopes—and flexible—fiberoscopes) are often used to inspect hard-to-reach places). Advanced optical measurement systems are increasingly used to measure the cracks and track their development on real objects (DIC-digital image correlation) [56].

More and more often, a scanning electron microscope (Hitachi Group, Dusseldorf, Germany) is also used to analyse the defects in the microstructure of concrete (SEM). SEM tests are very accurate but require special preparation of samples taken from the structure; thus, SEM tests are associated with slight damage to the structure. Therefore, these tests should be classified as semi-destructive tests. On their basis, it is possible to identify the causes of cracks [57,58].

An inventory of cracks allows one to estimate the causes of damage, locate places of potential weakening of elements, identify possible corrosion of the reinforcement and indicate the areas from which material for laboratory tests should be taken.

Electrochemical tests are particularly valuable because of the possibility of conducting them in the field with simultaneous assessment of the interaction of the current steel and concrete properties in real operating conditions. They make it possible to determine areas exposed to corrosion on the surface of elements as well as the degree of reinforcement corrosion advancement and to forecast its progression over time [10,11,16]. These tests are performed using specialised devices that allow the measurement of appropriate electrical quantities, the values of which indicate the course of the corrosion process on the reinforcement [1,10,14]. Currently, the most commonly used methods are mainly the potentiometric method, half–cell potentials (measurement of stationary potential and its gradient) [59], the polarisation method—measurement of corrosion current density [60,61,62,63]—and the resistance method [14] (concrete resistance—measurement of concrete cover resistivity). Currently, in the available literature, the most frequently described research is conducted using the half-cell potential method, although the experience of the authors and other researchers shows that this method is less reliable than the polarisation method (e.g., galvanostatic pulse method). In the paper [64], the authors presented the results of chemical tests as well as the measurements by the use of the galvanostatic pulse technique indicating an advanced corrosion process of the reinforcement in the elements of the bridge structure, while the half-cell potential method did not show it. The authors of this paper repeatedly drew similar conclusions from their research [23,29,30].

Among the above-mentioned electrochemical methods, the most reliable, and at the same time that which allows one to conduct measurements in the field in a relatively uncomplicated manner, are the measurements of the corrosion current density.

Polarisation methods, depending on the method of initiating the disturbance, are three in number: the electrochemical impedance spectroscopy method (disorder results from the action of alternating current in a wide frequency range); the linear polarisation resistance method (disorder is generated by applying a linearly changing potential); and the galvanostatic pulse method (the disorder is generated by a current with a certain intensity value).

In the case of diagnostic tests, it is worth using various measurement techniques simultaneously to verify the obtained results. It is particularly important to combine research with electrochemical methods with material tests and appropriate control opencasts.

The research described in the article used the GP-5000 GalvaPulse™ (Force Technology, Brøndby, Denmark) set for measurements using the galvanostatic pulse polarisation method. The equipment allows one not only to measure the corrosion current density (which allows one to estimate the corrosive activity of the reinforcement and forecast its rate), but also to measure the stationary potential of reinforcement and the resistivity of the concrete cover. The scope of the research makes it possible (on the basis of an analysis of the conditions conducive to corrosion) to determine the areas on the structure where the probability of corrosion is relatively higher than elsewhere. The obtained results are analysed in relation to certain criterion values, which in the form of tabulated data are attached to the apparatus (Table 1). Depending on the obtained values of the stationary reinforcement potential and the resistivity of the concrete cover, it is possible to conclude on the value of the corrosion probability of the reinforcement in the tested area. However, these are not always reliable measurements. On the basis of the value of the corrosive current density, its corrosive activity can be estimated and its rate forecast over time. At the same time, it should be remembered that the criteria presented in GP-5000 GalvaPulse™ set cannot be used elsewhere. When performing measurements with other equipment (due to a different type of reference electrode or a different way of polarisation of the reinforcement), the reference criteria are different.

A reinforcement covered in concrete is protected against corrosion by the steel passivation mechanism. However, it is only effective when the rebars are well-covered by concrete. Concrete may be affected by the environment and change its properties overtime. The carbonation process is considered to be very unfavourable. The effect of carbon dioxide that penetrates the concrete dissolves in the pore solution, resulting in the formation of carbonic acid. This acid reacts with the cement paste alkaline components, which results in the formation of carbonates and a lowering of concrete pH. When the pH drops below a certain level (pH < 11.8), the steel depassivates and in the presence of water and oxygen begins to corrode [21]. Carbonation, however, is a slow process. By detecting it at an early stage, it can be countered. For this reason, test methods that allow for quick and easy diagnosis of the carbonation process in concrete are being developed. Each of them has its own advantages and disadvantages. The advantages and limitations of many methods have been discussed by Qiu [22].

The most common methods for determining carbonation are colourimetric tests. They consist in spraying a solution on the tested concrete surface. Solution in contact with the concrete, depending on its pH, causes its colour to change according to its interaction with the surface. The most popular indicator used for this purpose is phenolphthalein. The study of carbonation with the use of phenolphthalein indicator is recommended by EN 13295:2004 and RILEM CPC-18. In this study, phenolphthalein, in contact with non-carbonated concrete with a high pH, causes its colouration to a characteristic, intense magenta colour. As a result of carbonation, the concrete pH may drop below 9. In this case, phenolphthalein produces no colouration of concrete. Based on this, carbonated and uncarbonated concrete can be distinguished. The determination of the concrete surface condition itself is unsatisfactory for diagnostic purposes. In tests, it is usually important to check the extent of carbonation in the concrete, which requires slight structural damage to collect a sample. This sample can then be used for other tests. However, due to the necessity to take samples from the structure, it is not a test used for continuous control of its condition. The limitations of this method also result from the phenolphthalein specificity, which can evaporate from the concrete surface in the period between the application and the measurement. As a result, the phenolphthalein concentration can change, which may affect the concrete colouration [22]. Time plays an important role in this type of test because of the individual phases of the cement matrix of concrete colour at different rates [66]. Phenolphthalein changes the concrete surface colour at a pH range from 8.5 up to 9.5. Meanwhile, depassivation of steel occurs when the pH drops to 11.5–11.8 [10,21]. For this reason, in colourimetric tests other indicators are also used, e.g., timoloftaleine, alizarin yellow R, alizarin yellow GG, or T^F^PLPt [22,67]. Thymolphthalein changes colour at a pH of 9.3–10.5, while T^F^PLPt can be used for pH tests in the range 11.0–13.5. The disadvantage of tests with phenolphthalein and thymolphthalein is the colouration of concrete to one colour only. As a result, they perform well in determining the depth of carbonation, but it is difficult to use them to determine its degree of advancement in particular areas. For this reason, colourimetric tests are currently being developed that also allow the identification of the pH distribution on the tested concrete surface. This type of test is the rainbow-test, which allows one to perform pH tests in the 5 to 13 range [10,22,68,69]. In this test, each change in pH by 2 is associated with a significant change in the obtained concrete colour. Concrete that is considered non-carbonated has the violet colour characteristic for pH = 11. Similarly, when a T^F^PLPt indicator is used, the different colours are obtained depending on the pH of the concrete being tested [67]. In this case, non-carbonated concrete turns greenish or cream-coloured. In the case of this indicator, it is possible to perform more accurate analyses of the pH distribution on the tested surfaces using a camera with appropriate band-pass filters.

Compared to other tests, the colourimetric test’s advantage is the speed and the ease of obtaining information about the pH lowering, which is a factor directly responsible for steel depassivation. More complicated but more precise tests of concrete pH concern the tests of the pH of the pore solution [10]. For this purpose, the tested concrete must be crushed to a particulate in the range of 0–0.2 mm, and then a model liquid must be obtained from such material by preparing a water extract or by vacuum or pressure extraction methods. The easiest way is to prepare a water extract by mixing ground concrete with distilled water in a 1:1 ratio and storing it for 24 h. After this period, the solid phase is filtered off and the pH of the liquid is measured with a pH-meter. The solution obtained in this way, however, does not fully correspond to the liquid that occurs naturally in the pores of concrete, as the pores are under a pressure other than atmospheric. A more accurate representation of the pore liquid is obtained by performing a vacuum extraction. For this purpose, after preparing a water extract of ground concrete, it is concentrated under vacuum. The best material for research is obtained using pressure extraction. During this, the test liquid is obtained by squeezing it from the concrete sample in a special press, applying a pressure of 450–500 MPa. In this way, small amounts of test liquid are obtained. By testing the pore liquid, one can measure the pH more accurately, but it is more difficult to determine the depth of carbonation, which requires many tests on concrete samples taken from greater and greater depth.

Computer X-ray tomography is a modern test that allows one to determine the carbonation front progress (XCT) [70,71]. Using this method, it is possible to test samples that do not require significant processing, and it is not necessary (e.g., as in colourimetric methods) to expose the tested surfaces. Carbonation measurement with this method is based on the fact that as a result of carbonation and the formation of calcium carbonate, the density of the concrete increases and its porosity decreases, which means that it absorbs more radiation [21,70]. As a result of this test, it is possible to determine the depth of both carbonated and uncarbonated concrete as well as its porosity, and to distinguish cracks in the concrete. In the case of reinforced concrete, it can also determine the location of the bars and determine whether they are not subject to corrosion as evidenced by a layer of rust, with a lower density and cracks around the bars.

Another test utilising X-rays that is used in testing the durability of concrete is X-ray diffraction (XRD). It can be used to determine the phase composition of concrete. Carbonated cement paste in concrete is characterised by reduced compactness and/or evident absence of portlandite. In this pastes in the place of portlandite various calcium carbonates are formed [28,66,72,73,74,75]. As a result of carbonation on the surface of the portlandite, amorphous calcium carbonates are formed, which over time turn into vaterite, from which aragonite can be formed. Ultimately, as a result of polymorphic transformations, calcium carbonate is produced in the form of calcite. In this form, calcium carbonate is often present in concrete as a component of aggregate, but it will not be present in the form of vaterite and aragonite. Thus, the detection of one of the metastable polymorphic forms of calcium carbonate in the course of research may indicate the ongoing process of carbonation. Such tests can be performed on small fragments of crushed concrete or cement paste matrix separated from it, that had been taken while performing other tests. For testing purposes it is also worth collecting drill cuttings, taken from different depths of concrete, in order to determine the depth to which the cement paste has been carbonated (using the Profile Grinder kit; (Force Technology, Brøndby, Denmark). However, testing of samples in which the cement paste is mixed with aggregate is very difficult, because the diffraction pattern is usually dominated by the aggregate peaks and it is difficult to identify the peaks originating from the cement matrix phases.

Fourier transformation infrared spectroscopy (FTIR) is also used to study the changes in the phase composition of the cement paste undergoing carbonation [22,72]. It can also be used to study the transformations of portlandite and the C–S–H phase as well as the CaCO_3_ polymorphism. In addition to being able to determine the amount of the resulting carbonation products, one can also study its depth. In order to test the carbonation depth, the peak for C–O bonds corresponding to a wavelength of 1415 cm^−1^ is measured. This peak decreases until a constant amplitude is attained after reaching the depth at which the carbonation occurs. The results of the molecular structure studies undertaken with FTIR testing provide more accurate information about the partially carbonated zone than the results of the phenolphthalein indicator test.

Infrared spectroscopy studies can be combined with other types of research, thus obtaining more accurate information about the composition of the carbonated paste. They can be complemented with XRD analysis [72,73], but can also be combined with thermal analysis [76]. Thermal analysis by the thermogravimetric (TG) method is a popular method that allows one to quantify the cement paste carbonation degree. It is based on the measurement of the sample weight loss during its heating. By taking samples from different depths of concrete, it is possible to determine the exact content in them of Ca(OH)_2_ and CaCO_3_, and thus it can be stated to what extent the former has transformed into the latter. Using this technique, it is also possible to study CaCO_3_ formed as a result of carbonation of the C–S–H phase, because crystallisation degree and thermal stability of the carbonates formed in this way is reduced [22]. However, it is not possible to distinguish polymorphic forms of calcium carbonate in concrete by thermal analysis. For this reason, this analysis is often supplemented with the phase composition analysis using the XRD or FTIR method.

**Figure 1:**
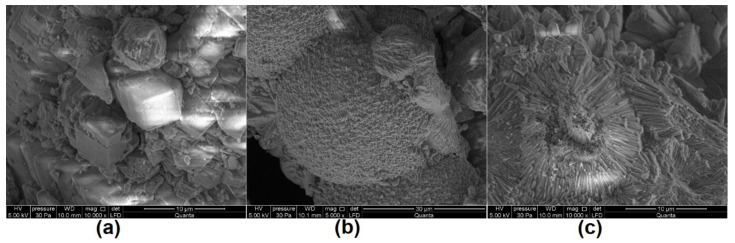
Calcium carbonates in carbonated drips on concrete described in [28]: (**a**) calcite, (**b**) vaterite, (**c**) aragonite.

Microstructural studies using a scanning electron microscope (SEM) or a transmission electron microscope (TEM) are often performed as complementary studies to phase analyses [22,28,72,74,77]. By observing the microstructure, changes can be observed on the surfaces of portlandite crystals as a result of the carbonation process [71] in the form of visible carbonation products (Figure 1). Thus, it is possible to estimate qualitatively to what extent the concrete is carbonated. Because of the differences in the porosity of the carbonated and non-carbonated paste [21,75,77], when conducting research on specimens at low magnifications, the depth of carbonation can also be determined. In such a study, Rimmelé et al. [78] were able to distinguish four successive zones of cement paste: non-carbonated core inside the paste, dissolution front, carbonation front and carbonated zone. These tests, as with those performed with the computed tomography method, allow for the location and assessment of the condition of the reinforcement. For SEM tests in this area (as opposed to XCT tests), samples should be specially prepared—they should be made into a polished section or thin section.

As a result of the analysis of the phase composition using the XRD, FTIR and DTG methods as well as the observation of the microstructure, it is possible to detect other substances potentially dangerous for reinforced concrete. Because of the possible corrosion of the reinforcement, attention is drawn to the presence of Friedel’s salts and other compounds containing chlorides. The presence of chlorides may cause the degradation of the cement paste, reducing its passivation properties, as well as having a significant impact on the corrosion of the reinforcement.

In order to study the progress of concrete carbonation, other spectroscopic methods can also be used: Raman, nuclear magnetic resonance and electrochemical impedance [22,79]. Carbonation and corrosion of the reinforcement can also be monitored using piezo-impedance transducers [80] or by measuring the absorption of gamma radiation [81].

## 3. Assessment of Selected Elements of the Historic Water Tower Structure

The following part of the article contains a description of the research aimed at assessing the condition of reinforcement and concrete cover in selected reinforced concrete elements of a historic water tower structure. The galvanostatic pulse method was used to assess the risk of reinforcement corrosion in concrete and forecast its rate over time in the structure elements, as well as X-ray diffraction and thermal diffraction analysis of the concrete cover. The ferromagnetic detection system was used to locate reinforcement in the structure elements.

### 3.1. The Research Methods and Used Materials

**Figure 2:**
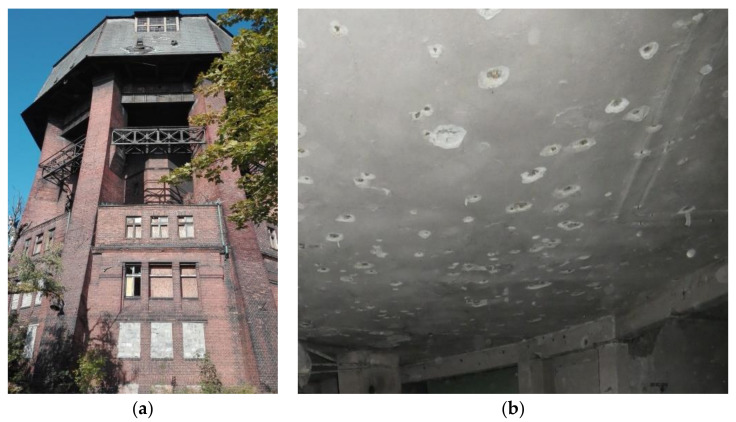
The picture of a historic water tower: (**a**) picture of the object, (**b**) pop outs in the concrete cover.

The water tower located in Zabrze (Silesia, Poland) was designed by an architect, August Kind, and Friedrich Loose, a building advisor. It was erected in 1909. The height of the building with respect to the ground level is about 45 m. The building has a unique construction (Figure 2a). It is based on an octagonal plan (the diameter of the base is 23 m) with 9 brick pillars—8 in the corners and 1 central. The pillars support the tank located at a height of about 28 m topped with a mansard roof. Between the pillars, in addition to one underground storey, there are three storeys accommodating a residential and office area.

At the end of 2018, an assessment of the tower’s structure was carried out as part of the reconstruction, extension and modernisation strategy scheduled in the project to revitalise it for social, educational, scientific and cultural purposes. During the inventory, numerous concrete cavities were observed on all reinforced concrete elements of the supporting structure in the residential and office areas (Figure 2b). For this reason, it was decided to assess the state of reinforcement and concrete cover based on carefully selected tests.

**Figure 3:**
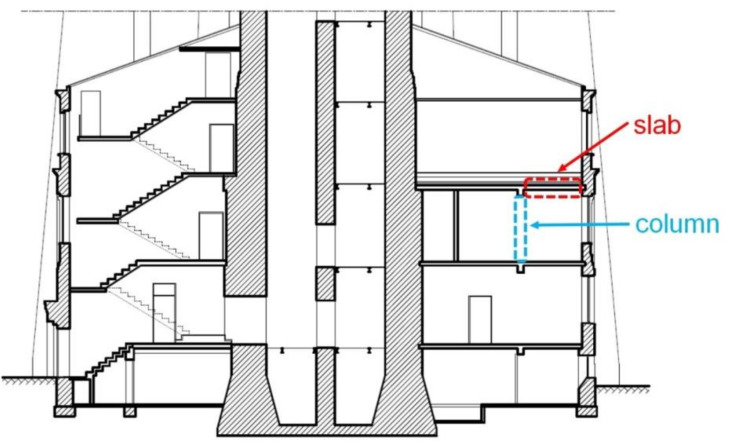
The vertical cross-section of the water tower lower part with measurement locations—the plan sketch.

Because of the historic character of the building, only non-destructive and semi-destructive testing were possible. A building inventory was developed and the structural system of the building was identified. Two elements were chosen for detailed diagnostic tests: a ceiling slab above the second floor and a load-bearing column (Figure 3).

**Figure 4:**
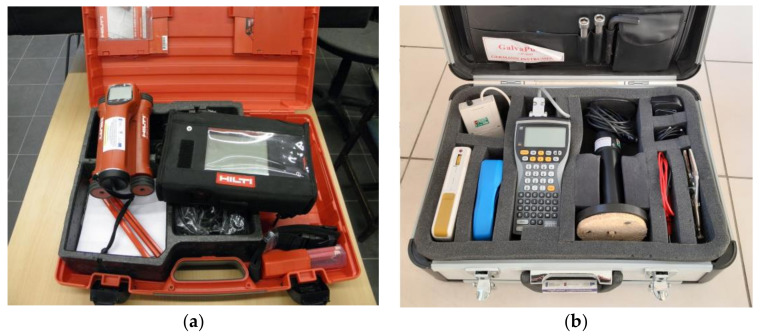
Measuring devices: (**a**) PS 200 Hilti scanner, (**b**) GP-5000 GalvaPulse™ kit.

The testing was carried out in two stages. In Stage I, in situ tests were performed to determine the rebar location and layout, rebar corrosion degree and the carbonation depth in the concrete cover. Samples were taken for laboratory tests. Stage II included laboratory tests of the concrete cover. The Hilti PS200 scanner was used to locate reinforcing bars in the concrete (Figure 4a). Software added to the device helped determine the thickness of the concrete cover. The device operates on the principle of electromagnetic induction [10,82]. The measurements helped to assess deviations in the concrete cover thickness in the tested areas. Thickness deviations can seriously affect the protective properties of the concrete [15].

The degree of reinforcement corrosion was evaluated via the electrochemical galvanostatic pulse method (GPM) [13,23,65]. This measurement method is an alternative to the more commonly used half-cell potential measurements. The commonly utilised potential mapping technique, measuring the half-cell potential on a concrete surface, sometimes has led to misinterpretation. For this reason the GPM has been introduced as a more advanced technique. Both electrochemical methods assume that corrosion of reinforcement in concrete is an electrochemical process. A steel bar serves as an electrode and the alkaline liquid filling in the concrete pores as an electrolyte. Local anode and cathode microcells formed on the surface of the bar generate the flow of electric charges through the bar, while the liquid filling the concrete pores carries the ions. Measurements are taken along the bar on the concrete surface after connecting the device to the bar. It is therefore necessary to expose the rebar along a few centimetres of its length. In the galvanostatic pulse method, the short-time anodic current pulse is impressed galvanostatically, which leads to the polarisation of the reinforcement and allows one to measure certain electrical quantities, i.e., the reinforcement’s stationary potential (E_st_), corrosion current density (i_cor_) and concrete cover resistivity (Θ). The obtained values, after comparing them with the criterion values (Table 1), allow assessing the reinforcement corrosion degree in concrete. In these tests, the GP-5000 GalvaPulse™ set was used to make measurements (Figure 4b). The main elements of the set include the control and recording device (PSION minicomputer), silver-chloride reference electrode (Ag/AgCl) and calibration device. The advantage of using the GalvaPulse™ set is the relatively short measurement time—usually no more than thirty seconds at any given point.

Depth of carbonation into the concrete cover at the sites of exposed rebar was determined via the carbonation test (1% phenolphthalein solution test) [83].

Stage II included laboratory tests of the concrete cover samples extracted from the structure. X-ray diffractometry (XRD) and thermal analysis (DTA-TG) methods were used [84,85]. The phase composition of the samples was identified by X-ray diffraction in an Empyrean diffractometer (PANalytical, Almelo, The Netherlands) equipped with a Cu lamp and a X’Celerator silicon detector. The analyses were carried out in the range from 5 to 75°θ, and the ICDD PDF-2 database was used to interpret the diffractograms. The thermal analysis was performed in STD Q600analyser (TAinstruments, Hüllhorst, Germany). The measurements were carried out in a nitrogen atmosphere increasing the temperature to 1000 °C at a rate of 10 °C/min.

### 3.2. Tests Results

#### 3.2.1. Reinforcement Location and Concrete Cover Thickness Measurement

**Figure 5:**
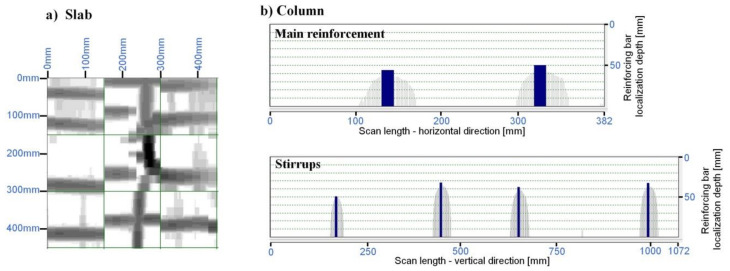
Image of reinforcing bars location in concrete elements, obtained with the use of Ferroscan device.

The rebar position and layout in the elements were determined with the Ferroscan detector. For this purpose, a 3 × 3 reference grid with a cell size of 0.15 × 0.15 m was attached to the concrete surface in the selected site on the slab (Figure 2). This was followed by running the scanner along the grid rows and columns (“Imagescan” mode). The location of the rebars helped select the site for concrete cover removal for testing. The scans and measurements carried out on the exposed rebars found that the main reinforcement of the slab was composed of square bars of 7.5 mm side-spaced about 8–15 cm apart. The software (PS 200 Software, release 5.4.2.1) included in the device, after entering the data from the initial rebar diameter measurements, measured the thickness of the main rebar coverage at twenty points. On the basis of the obtained concrete cover thickness values, the minimum, maximum and average values as well as standard deviation and the variability index were determined. Obtaining a large number of such scans of the structure and the statistical analysis of the results allows to find places with the smallest cover thickness and the greatest dispersion of values. Corrosion of the reinforcement in these places is most likely due to the fastest destruction of the concrete cover. The results are compiled in Table 2. The graphical representation of the rebar detection results is shown in Figure 5a.

The rebar diameter measurements found that four square bars with 14 mm cross-section were used as the main reinforcement of the column. Because of the small width of the column, scanning was performed using the “Quickscan” method. The results of concrete cover thickness measurements are presented in Table 2. The graphical representation of the rebar detection results is shown in Figure 5b.

#### 3.2.2. Degree of Reinforcing Bar Corrosion

**Figure 6:**
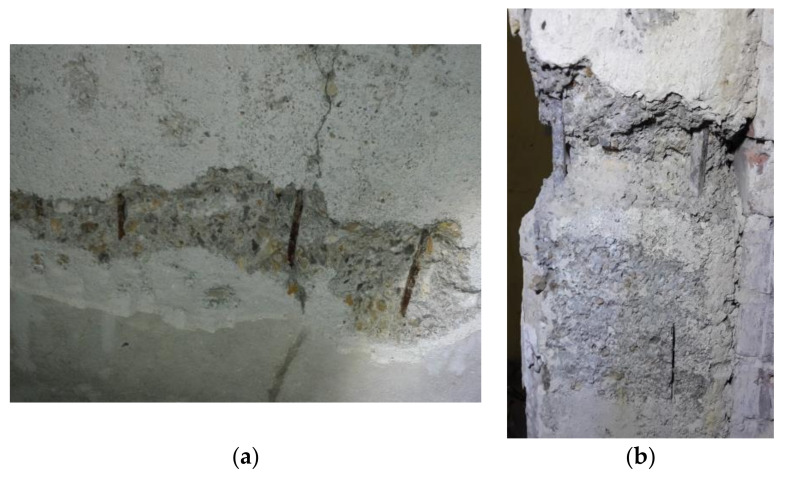
(**a**) Floor slab with exposed reinforcing bars; (**b**) column with exposed section of the bar under test.

Because of the visible poor condition of the concrete and the concrete cover carbonation confirmed by in situ tests, it was necessary to assess the state of reinforcement in the structure. The degree of reinforcement corrosion was assessed in the selected areas of the floor slab and column (Figure 3). Four bars were accessed in the floor slab (Figure 6a). Six points for measurements were planned to be set on the concrete surface along each bar (at ~10 cm spacing), that is to say, a total of twenty-four measurement points were established. Unfortunately, for an unknown reason, all rebar no. 1 measuring points specified by coordinates (1.1)–1.6) failed to provide results useful for analysis. Ultimately, the results from eighteen measurement points were analysed. In the column, two main reinforcing bars were accessed at a height of about 1.0 m from the floor level (Figure 6b). Along these bars, four points on each of them were determined at a spacing of ~10 cm, which gave a total of eight measurement points.

The measurement results of all three parameters (E_st_, Θ, i_cor_) from the GP-5000 GalvaPulse™ device, measured at individual points, are summarised in Table 3 (slab) and Table 4 (column). The values of the predicted corrosion rate [μm·year^−1^] and maps of the distribution of values of all measured parameters were generated in the PsiWin program (included in the GP-5000 GalvaPulse™ set), Figure 6a for the floor slab and Figure 6b for the load-bearing column.

The results were analysed based on the criteria presented in Table 1, which allowed determining the probability of rebar corrosion in the areas that were examined as well as assessing the corrosion degree and estimating the rebar corrosion rate.

**Figure 7:**
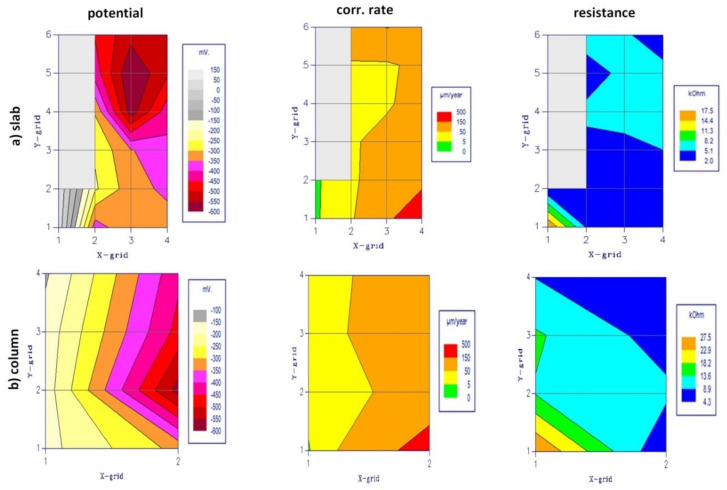
Measurement results of galvanostatic pulse method.

In the floor slab, the analysis of the stationary potential of the reinforcement showed that the probability of corrosion in the selected area of the slab was very high. At ten measurement points, it exceeded E_st_ = −350 mV, which indicates a 95% probability of corrosion. At the remaining eight points, a 50% probability of reinforcement corrosion was obtained. The results of concrete cover resistivity also indicate a high probability of reinforcement corrosion, because at all eighteen points the values were lower than Θ = 10 kΩ·cm (Figure 7). Measurements of corrosion current density indicated the ongoing corrosion process of the bars that were tested, although the intensity of this process varied. Low corrosion activity (i_cor_ = 2–5 μA·cm^−2^) was obtained for rebar no. 2 and two points of rebar no. 3. Moderate corrosion activity (i_cor_ = 5–15 μA·cm^−2^) was observed at four points of rebar no. 3 and five points of rebar no. 4. High corrosion activity was found only at one point of rebar no. 4, where the corrosion current density exceeded i_cor_ = 15–A·cm^−2^. At the same time, the results allowed estimating the corrosion rate in the tested rebars within the 33–57 μm/year range in rebar no. 2, 40–153 μm/year in rebar no. 3, and 68–182 μm/year in rebar no. 4, indicating a very poor condition of the floor slab reinforcement.

The same analysis was carried out for two reinforcing bars in the load-bearing column. A low 5% probability of corrosion (the values of the rebar stationary potential did not exceed E_st_ = −200 mV) was obtained at all four points of the rebar no. 1. But in rebar no. 2, the reinforcement stationary potential was much higher. At one point, rebar no. 2 indicated a 50% probability of corrosion, and at the other 3 points the corrosion probability was 95% (E_st_ < −350 mV). The values of concrete cover resistivity in rebar no. 1 indicated the ambiguous probability of corrosion, from small (at one point, Θ > 20 kΩ·cm) through medium (at two points, 10 < Θ < 20 kΩ·cm) to large (at one point, Θ < 10 kΩ·cm). In rebar no. 2, the occurrence of corrosion probability at all points was defined as large (Θ < 10 kΩ·cm). Measurements of corrosion current density of the column reinforcement were consistent with the measurements of the corrosion potential and resistivity of the concrete cover. At two points determining the corrosion activity in rebar no. 1, the measured values did not exceed i_cor_ = 2 μA·cm^−2^, which indicated that the activity was not predicted or negligible, and at the remaining two points, the values slightly exceeded i_cor_ = 2 μA·cm^−2^, which should be read as low corrosion activity. The rate of corrosion development in this rebar was estimated at 2.2–30.1 μm/year. However, at the points determining the corrosion activity in the rebar no. 2, the values of the corrosion current density were much higher, indicating a moderate (at three points, i_cor_ = 6.71–8.19 μA·cm^−2^) and even high corrosion activity in this rebar (at one point, i_cor_ = 17.53 μA·cm^−2^). The rate of corrosion development in rebar no. 2 can be estimated as 203.3 μm/year.

#### 3.2.3. Laboratory Testing of Concrete Cover

Field assessment of depth of carbonation into the concrete surface (carbonation test with a phenolphthalein solution) of the removed concrete cover pieces revealed complete carbonation of the concrete cover (no concrete colouration, indicating pH < 8.5).

**Figure 8:**
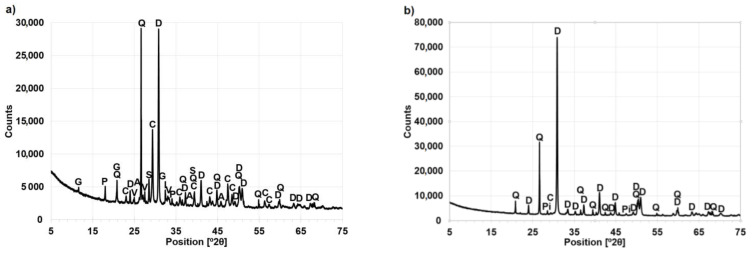
Phase analysis (XRD) of (**a**) cement paste, (**b**) aggregate recovered from the concrete. Notation: A—Aragonite, C—calcite, D—Dolomite, G—Gypsums, I—Ilmenite, Q—Quartz, P—Portlandite, Pi—Pyrite, S—Sylvite, V—Vaterite.

Laboratory tests of the concrete samples show that as a result of the carbonation process, the paste underwent significant changes. Three polymorphic forms of calcium carbonate (CaCO_3_): calcite, aragonite and vaterite are visible in the X-ray diffraction pattern in Figure 8a [28,86]. Analysis of the used aggregate (Figure 8b) reveals the presence of only a trace of calcite. Despite intense indications from CaCO_3_, portlandite peaks are still clearly visible in the sample. Analysis of the paste sample confirms the presence of a significant amount of aggregate dust fractions, which is represented by the intense peaks of dolomite and quartz appearing on the X-ray patterns. The presence of dolomite dust contributes to noticeable loss of sample mass above 600 °C. This is also associated with the presence of carbonation products. Gypsum and sylvite found in the paste indicate that the concrete may have been exposed to sulphate and chloride attack. The aggregate tested consists of dolomite, which is accompanied by quartz mixed with pyrite (Figure 8b).

**Figure 9:**
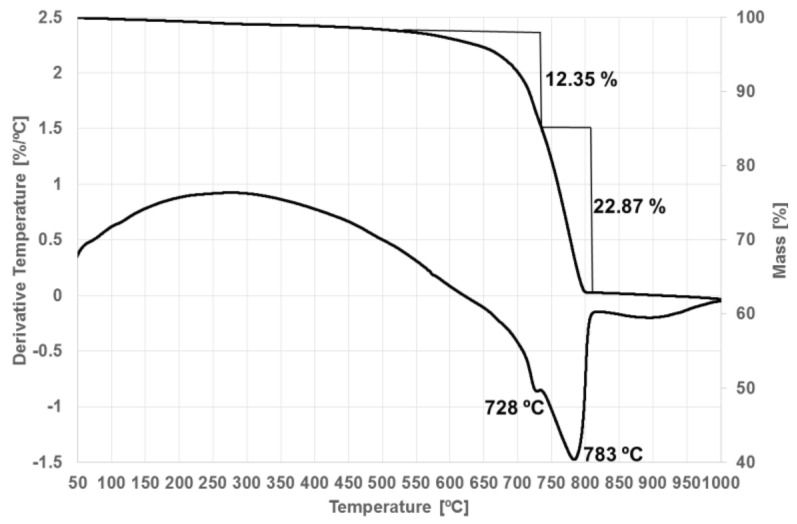
Thermal analysis of aggregate.

The results of the X-ray analysis of the aggregate are supported by the results of the thermal analysis. Analysis of the DTA curve of the aggregate (Figure 9) shows a double endothermic effect characteristic of dolomites [87,88], whose first maximum (728 °C) is associated with the thermal decomposition of dolomite, and the second (783 °C) with decarbonation of the resulting calcite. At the same time, the DTA curve shows a very low endothermic effect at 573 °C associated with the polymorphic transformation of low-temperature α-quartz into high-temperature β-quartz.

The phase composition analysis allows for precise recognition of effects carbonation. Based on the analysis, the possibility of the presence of calcium carbonate in concrete as a result of its introduction with carbonate aggregate was excluded. It has also been established that CaCO_3_ is found in the 110-year old concrete both in stable calcite form as well as vaterite and aragonite. As a result of X-ray analysis, it was found that the degradation of concrete caused by carbonation could additionally be caused by the effect of sulphates.

## 4. Conclusions

Diagnostics of historic objects is usually associated with limitations resulting from the possibility of using only non-destructive or semi-destructive methods and the possibility of taking a small number of samples for material testing. However, the tests carried out on selected elements of the supporting structure of the historic 110-year old water tower indicate that they are sufficient for the initial assessment of the condition of reinforcement and concrete cover of the structure. Based on the local visual assessment supplemented with measurements of the concrete cover thickness, the locations of the remaining tests were easily determined. In situ concrete pH measurements allowed one to determine whether concrete still fulfils its protective role in relation to the reinforcement. However, only a full and thorough analysis of laboratory tests allowed the determination of the causes of corrosion. To obtain reliable results, tests should be carried out with several mutually verifying methods, such as electrochemical assessment tests of the reinforcement corrosion level, supplemented with laboratory tests of the concrete cover.

Performed research and analysis allowed the following conclusions to be drawn:

1. Non-destructive testing of the rebar location and layout performed using the Ferroscan gave accurate information on the location of reinforcing bars, their diameters and their cover thickness, all of which were positively verified after small pieces of concrete were removed.
2. Concrete cover distress was due to sulphate attack attributed to the presence of gypsum in the paste.
3. Non-destructive corrosion degree measurements of the reinforcing bars in the floor slab and in the load-bearing column determined the probability of corrosion in the examined areas, estimated corrosion activity in individual rebars, and predicted its pace over time. The results indicate a very poor state of the reinforcement in both the floor slab (high corrosion probability and locally high or moderate corrosion activity in the reinforcement) and column (high probability of corrosion and high corrosion activity in one of the two bars).
4. The carbonation test for in situ concrete showed total depassivation across the cover depth.
5. Material tests confirmed the information on the degree of concrete carbonation in the tested elements. Concrete carbonation was accelerated by the destruction of the concrete structure as a result of sulphate corrosion.

The described tests still need to be supplemented with diagnostic tests to assess the condition of the structure, mainly in terms of its load-bearing capacity. These tests should include, among others, determining the strength parameters of concrete and steel, crack morphology and displacement measurements. Because of the historical nature of the object, non-destructive and semi-destructive sclerometric, acoustic, pull-off methods can be used in the research.

## Figures and Tables

**Table 1:** Criteria for assessing the degree of reinforcement corrosion risk [65].

Criteria for Assessing the Degree of Reinforcement Corrosion Risk by Use the Galvanostatic Pulse Method				
Corrosion Current Density	i_cor_ [μA·cm^−2^]	Reinforcement corrosion activity		Corrosion pace; [mm·year^−1^]
		<0.5	not forecasted corrosion activity	<0.006
		0.5–2.0	Irrelevant corrosion activity	0.006–0.023
		2.0–5.0	Low corrosion activity	0.023–0.058
		5.0–15.0	Moderate corrosion activity	0.058–0.174
		>15.0	high corrosion activity	>0.174
Reinforcement Stationary Potential	E_st_ [mV]	<−350	95% of corrosion probability	
		−350–−200	50% of corrosion probability	
		>−200	5% of corrosion probability	
Concrete Cover Resistivity	Θ [kΩ**·**cm]	≤10	high corrosion probability	
		10–20	medium corrosion probability	
		≥20	small corrosion probability	

**Table 2:** Concrete cover thickness for the slab and column.

Average Value c [mm]	Maximum Value c_max_ [mm]	Minimum Value c_min_ [mm]	Standard Deviation s [mm]	Coefficient of Variation V [%]
Slab				
21.3	26	18	2.5	12
Column				
38	50	38	8	21

**Table 3:** Results from the measurements of the reinforcement stationary potential, corrosion current density and resistivity of concrete cover in the floor slab.

Meas. Points	Values			Meas. Points	Values			Meas. Points	Values		
	E_st_	i_cor_	Θ		E_st_	i_cor_	Θ		E_st_	i_cor_	Θ
(2, 1)	−376.06	3.53	4.90	(3, 1)	−307.06	12.23	2.10	(4, 1)	−324.14	15.71	2.00
(2, 2)	−242.30	2.98	4.00	(3, 2)	−327.65	8.82	4.70	(4, 2)	−362.29	12.02	2.60
(2, 3)	−256.27	3.65	3.10	(3, 3)	−346.85	6.07	4.10	(4, 3)	−384.76	6.83	5.10
(2, 4)	−304.25	2.81	6.40	(3, 4)	−575.50	3.66	6.40	(4, 4)	−422.67	6.50	6.30
(2, 5)	−421.74	4.23	2.30	(3, 5)	−596.33	3.44	6.60	(4, 5)	−508.80	5.84	6.80
(2, 6)	−453.80	4.92	8.00	(3, 6)	−533.84	13.19	5.90	(4, 6)	−535.95	10.74	2.00

**Table 4:** Results from the measurements of the reinforcement corrosion potential, corrosion current density and resistivity of concrete cover in the load-bearing column, storey II.

Meas. Points	Values			Meas. Points	Values		
	E_st_	i_cor_	Θ		E_st_	i_cor_	Θ
(1, 1)	−18372	0.19	27.50	(2, 1)	−316.19	17.53	4.30
(1, 2)	−173.89	1.55	13.30	(2, 2)	−568.48	6.71	9.90
(1, 3)	−169.44	2.47	14.20	(2, 3)	−467.14	8.19	6.80
(1, 4)	−142.76	2.59	8.80	(2, 4)	−437.42	7.28	6.60

## Data Availability

No new data were created or analyzed in this study. Data sharing is not applicable to this article.
